# Rethinking treatment-resistant depression to quasi-tenacious depression

**DOI:** 10.1192/j.eurpsy.2022.2353

**Published:** 2023-01-12

**Authors:** Shokouh Arjmand, Rodrigo Grassi-Oliveira, Gregers Wegener

**Affiliations:** 1 Translational Neuropsychiatry Unit, Department of Clinical Medicine, Aarhus University, Aarhus, Denmark; 2 School of Medicine, Pontifical Catholic University of Rio Grande do Sul, Porto Alegre, Brazil

**Keywords:** Difficult-to-treat depression, quasi-tenacious depression, tenacity index, treatment-resistant depression

## Abstract

With almost one-third of patients with major depression not adequately responsive to treatments, the management of treatment-resistant depression (TRD) has continued to be challenging. Recently, an essential step was taken to replace TRD with difficult-to-treat depression (DTD), pointing to some drawbacks associated with this terminology and identifying addressable barriers. In line with the DTD concept, we discuss why terming this population of patients as TRD could be semantically and clinically misleading. We then suggest replacing TRD with quasi-tenacious depression (QTD), a model and terminology that are derived from a potentially measurable outcome, the tenacity index (TI). QTD predicts that in theory remission is achievable by providing suitable treatments at hand. QTD states that every patient with major depression (even those who respond well) has some degree of tenacity that needs to be overcome by the use of proper treatment modalities. Ergo, in patients with a higher TI, due to the dearth of available armamentaria, one might suffice to achieve a partial resolution of symptoms balanced with an optimal quality of life. However, QTD calls for an incessant pursuit of novel treatments and the identification of contributing factors leading to high TI. On a track toward personalized psychiatry, and in harmony with DTD, QTD embraces all key barriers leading to a failure to treatment response and tries to provide a measurable entity for a better clinical decision while conveying a dynamic positive outlook of the disorder for both patients and health care providers.

A map of the world that does not include Utopia is not worth even glancing at…Progress is the realization of Utopias.

Oscar Wilde

After decades of a tussle for improved management of depressive disorders, we still face challenges in the way of properly diagnosing and treating many individuals suffering from depressive disorders. Many patients with depression do not adequately respond to our antidepressant treatment tactics, and those who ostensibly respond sufficiently may experience either relapse or recurrence afterward [1]. Those “nonresponders or partial-responders” individuals are classified as patients with “treatment-resistant depression” (TRD), a semantically misleading terminology that could potentially disguise proper identification of the phenomenon. Though invaluable actions have been taken to improve the problems associated with this terminology [2, 3], and an alternative concept has been suggested [4–7], we still believe that there is room for improvement and in line with such endeavors, here, we propose a new concept hinged on the concept of difficult-to-treat depression (DTD) that could help to overcome some of the existing challenges in the field.

## A Poor Definition

Semantically, we argue that borrowing the term “treatment resistance” from infectious and neoplastic diseases is an inconsistent approach. It is not necessarily the same picture as is seen in depressive disorders; apart from the intrinsic resistance that predates antimicrobial agents and chemotherapy, resistance can be something that is evolved, and something that a biological system further acquires and develops over time to act more competent to escape various treatment modalities. It is not a one-off phenomenon, but something that can potentially be unquenchable as a coping mechanism. Moreover, the definition of TRD has relied on having at least two failed treatment responses (considering adequate dosing and optimal time course), which is a rich source of controversies and active debates [4, 8] (in Box 1 we have raised some questions that subsume several drawbacks of this terminology). Such ambiguities and shortcomings in fully addressing this group of people with depression keep us in a slough of unsatisfactory and insufficient management of several cases with depressive disorders and have hampered our progress toward a radical breakthrough.

Box 1. Open questions and drawbacks on TRD terminology

1. Who or what is resistant? The patient or the disorder?
2. What is a response, and what is an adequate response? Is there any way to quantify that? And to what extent can we rely on our currently available questionnaires and rating scales?
3. Is a treatment response adequate? Or should we also be taking remission, recovery, relapse, and recurrence into account? Could adding a second antidepressant after one failed trial of antidepressant lead to a colloquially “response,” the alleviation of a range of symptoms, but at the same time potentially give rise to a faster relapse rate?
4. According to the ACE model of mood disorders, different axes of symptoms resolve at different timeframes, cognition surpassing emotion and activity as the last component to respond to treatment or following spontaneous remission [9]. So, what do we count as treatment response, and how many axes must be resolved or remain impaired to call a patient “treatment responder” or “treatment-resistant,” respectively?
5. Where is the threshold between partial response and no response? Can a partial responder later undergo *metamorphosis* into a nonresponder?
6. Why do we need to have at least two failed trials of antidepressants and not three, or four, or five? Where a line should be drawn?
7. Which treatment strategy should be offered first and why? Could there be a different outcome envisaged that is influenced by selecting the first and second antidepressants?
8. What if the second tried-out antidepressant ameliorates a range of symptoms exhibited but below a subjective threshold for a response? What would be the next move?
9. Could the initial choice of an antidepressant affect the future response to any treatment?
10. For how long do we need to wait to follow up for a response? Does a delayed response to treatment necessarily indicate TRD?

Another issue is that almost all pharmacological armamentaria we boast in our arsenal have not targeted distinct biological systems since the initial discovery. It is not cogent to expect a “miracle” after a failure in response to almost similar treatments. Thus, we may need to ponder that the disease might not be resistant *per se.* With the advent of novel antidepressants with diverse mechanisms of action, we might be able to see a significant portion of TRD patients respond well to the treatment, as it often is the case for multitargeted interventions, such as electroconvulsive therapy, and ketamine.

To move one step further and come up with a solution, we here suggest supplanting this term with *quasi-tenacious depression (QTD)* that, unlike TRD or DTD, does not convey a negative impression and suggests a dynamic phenomenon.

In this model, basically, there is no absolute resistance or difficulty, but a degree of tenacity could be depicted as a ground that renders approaching the disorder more appropriately.

Besides, those who respond well to the treatment or spontaneously remit also have some degree of tenacity.

*QTD* implies that sometimes the disorder may seem tenacious, but in reality, it is not. The current approach to diagnosing patients is solely based on descriptive data (psychiatric interviews with patients and their relatives and clinical observation), rather than quantifiable measures (brain scans, blood samples, etc.), that specifically relate to the etiology and pathology of the underlying disorder. Therefore, differentiating between whether the complexion is the actual manifestation of a mental illness or the consequence or the effect of another masked etiology is intricate. As a consequence, the tenacity observed could be a result of either misdiagnosis, the true nature of the disorder, a personalized manifestation of the disease itself, patients’ pharmacogenomics, improper choice of pharmacotherapy, lack of therapeutic interventions, patients being nonadherent, protracted presence of a stressor (traumas, social-related contexts, dysfunctionalities, etc.), accompanied possible comorbidities (both somatic and psychiatric), patient’s school of thoughts, the currently used rating scales, or finally the disease classification system that might intrinsically lead to a misconstrued diagnosis [7, 10].

## A New Paradigm

We propose a new concept, the *tenacity index (TI)*, which can be defined as a magnitude of potential barriers and the degree to which a depressed patient might “seem” tenacious to respond to a particular treatment strategy to get a treatment response (an aspiration that is theoretically always achievable). Importantly, it is of note to mention that for the sake of clarity and simplification we have used, in the following, an analogy, and we, by no means, intend to compare a chemical reaction with a psychiatric illness.

**Figure 1.**
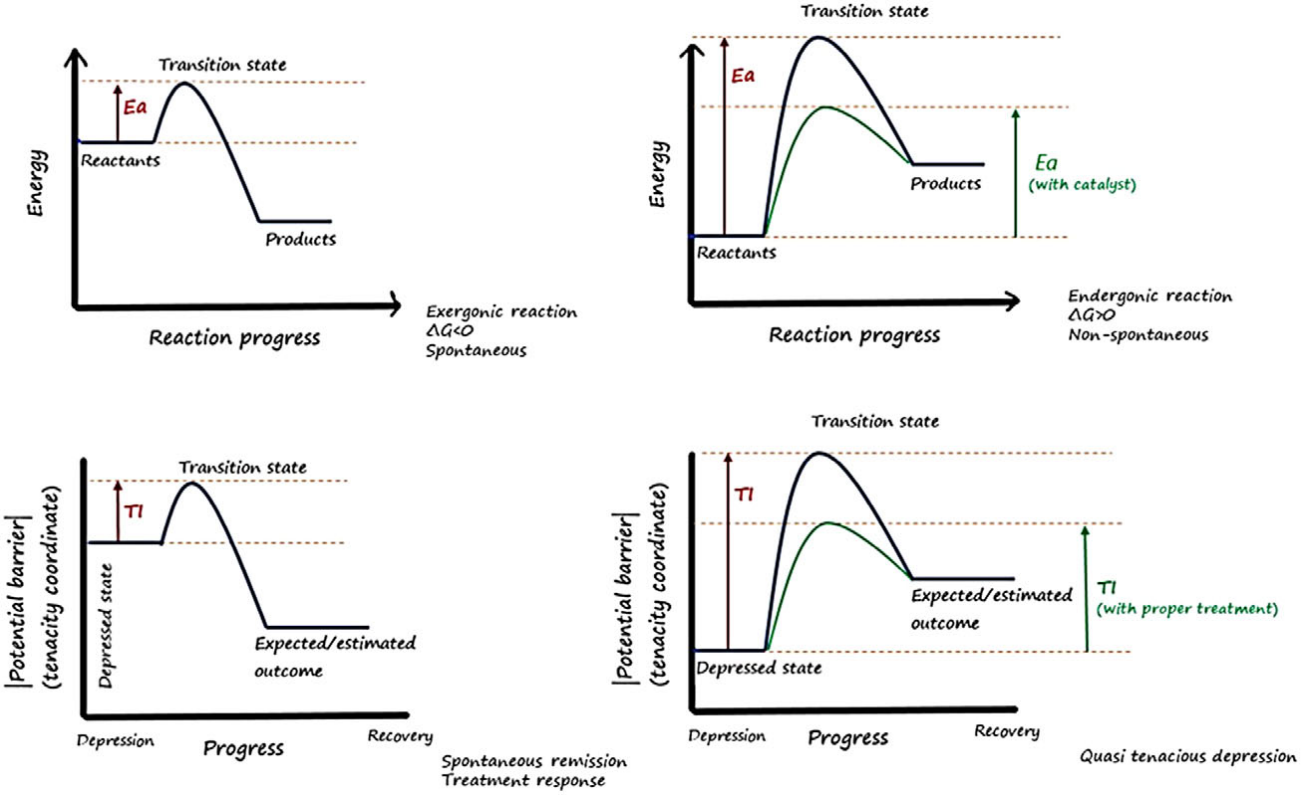
A schematic analogy between the tenacity index and activation energy for elucidation of the concept.

Using this concept, as depicted in Figure 1, the TI can be analogized to the *activation energy (E_a_)* of a chemical reaction describing the amount of energy required to reach the *transition state.* Accordingly, the current status of a patient in the depressed state can be analogized to the *reactants* and the patient’s estimated/expected resolution status to the *products.* The transition state theory states that once the reactants pass through the transition state, the reaction continues to make products. In this analogy, *QTD* is like *endergonic reactions* where the *E_a_ (TI)* is high, and to attain the transition state where remission is likely to be gained, use of the best suitable *catalyst* (proper use of armamentaria) to transcend the *E_a_ (TI) is needed.* As each patient is a unique individual *(having different initial state energy),* it is, therefore, necessary to set the ambition to a level to be able to overcome the *TI,* in order to achieve a complete remission and recovery with no functional and cognitive impairments. Theoretically, these Utopias are all attainable but the treatment strategy (*catalyst)* needed to be employed in order to push the reaction along by reducing the *TI* (*E_a_*), matters the most. Like what is observed in *exergonic reactions (less E_a_),* a patient can be seen either remitting spontaneously or responding straightforwardly to an antidepressant. However, it is noteworthy that akin to a catalyst that can only reduce the *E_a_* and not change the initial and final energy states of the reactants and products, a chosen treatment strategy merely dwindles the *TI.* It can alter neither the initial state of a depressed patient nor the final state of a treatment outcome, so it is considered a state function. It is also conferred that, unlike the treatment guidelines, in case of approaching a patient with a high index of tenacity, it is not necessary to wait for two failed treatment trials to start the available last resort of pharmacotherapy, given that the *TI* (*E_a_)* is what that should be undertaken.

To complete this scenario, we would also like to add three more complementary factors into the equation: The rate to remission (*R_m_*), the rate to relapse (*R_l_*), and the ratio of *R_m_* to *R_l_* (RR). With these additional determinants, the likelihood of whether a patient is to relapse *(a shift of the equilibrium toward the opposite direction)*, how fast remission can be achieved, and how the process *(reaction)* best can be speeded up by using different treatment modalities *(catalysts)* through a cost–benefit approach are predicted. The equilibrium shift to the relapse side can be due to a change in the equilibrium conditions; it might not be entirely avoidable but should be predicted and counteracted to reduce its effect.

We suggest that the *TI* can be a valuable concept to quantify the heterogeneity of depression, predict the outcome of a particular treatment, and improve treatment choice. To measure or estimate it, attention to the vital contributing elements needs to be given in a systematic manner, for example, the age and body surface area of the patient, the age at the onset of the disease, the duration, and pattern of the episodes, the chronicity of the disorder, core symptoms exhibited, the severity of each affected domain, the advent and the order of appearance of symptoms manifested and resolved, possible general medical comorbidities, possible psychiatric comorbidities, the severity of the disorder, genetics, and pharmacogenomic features, prior response to different treatment options (pharmacological/psychological/brain stimulation), the likelihood of polypharmacy, presence of persistent functional impairment even after remission, depression in the context of bipolarity, exposure to early life stressful events or trauma, depression subtypes and specifiers (atypical, melancholic, mixed, anxious, etc.), familial history, history or current suicidal ideation/attempts, and self-mutilation are some examples [7, 10].

## Clinical and Preclinical Upshots: What Needs to be Done?

Though the concept of QTD is only some initial thoughts, we believe that such an amendment may leave a significant impact. In a clinical context, it is one step closer to personalized psychiatry. *QTD* transfers a positive and flexible idea to the patients and healthcare providers. It promulgates that even full recovery is achievable with an apposite predictive tool and a proper treatment strategy. In patients with higher *TI,* since we currently want for a breadth of armamentaria to overcome that TI, we might suffice to achieve a partial resolution of symptoms balanced with an optimal quality of life (like the strategies already used in other disciplines of medicine, e.g., in the management of diabetes, neurological or inflammatory disorders). However, *QTD* underscores an incessant endeavor for a quenchless search and pursuit of the Utopia. In addition, *QTD* would not lead to diagnostic inflation [5] but instead would attempt to look at each patient as an individual in the broad concept of depressive disorders.

As a future perspective, comprehensive clinical studies could aim first to rule out the contaminating factors [6] and then attribute a value to the contributing elements of the *TI* [11] in prospective or retrospective studies. Such an endeavor might lead to a new perspective regarding our understanding of mood disorders. It also points to an essential need for a dynamic framework to improve our classification system (categorizing patients based on their *TI*) and treatment strategies (setting about a treatment modality hinging on the *TI*) toward refining available guidelines. Such slant helps enroll more homogenous samples of depressed patients in the clinical trials by quantifying the determinants and emphasizing the Research Domain Criteria (RDoC) approach; hence it can facilitate the transition of research “from bed to bedside.”

## References

[1] Cuijpers P. The challenges of improving treatments for depression. JAMA. 2018;320:2529–30. doi:10.1001/JAMA.2018.17824. 30500053

[2] Sforzini L , Worrell C , Kose M , Anderson IM , Aouizerate B , Arolt V , et al. A Delphi-method-based consensus guideline for definition of treatment-resistant depression for clinical trials. Mol Psychiatry. 2021;27:1286–99. doi:10.1038/s41380-021-01381-x. 34907394 PMC9095475

[3] Howes OD , Thase ME , Pillinger T . Treatment resistance in psychiatry: state of the art and new directions. Mol Psychiatry. 2021;27:58–72. doi:10.1038/s41380-021-01200-3. 34257409 PMC8960394

[4] Rush AJ , Sackeim HA , Conway CR , Bunker MT , Hollon SD , Demyttenaere K , et al. Clinical research challenges posed by difficult-to-treat depression. Psychol Med. 2022;52:419–32. doi:10.1017/S0033291721004943. 34991768 PMC8883824

[5] Cosgrove L , Naudet F , Högberg G , Shaughnessy AF , Cristea IA . Reconceptualising treatment-resistant depression as difficult-to-treat depression. Lancet Psychiatry. 2021;8:11–3. doi:10.1016/S2215-0366(20)30416-8. 33065028

[6] McAllister-Williams RH , Arango C , Blier P , Demyttenaere K , Falkai P , Gorwood P , et al. The identification, assessment and management of difficult-to-treat depression: an international consensus statement. J Affect Disord. 2020;267:264–82. doi:10.1016/J.JAD.2020.02.023. 32217227

[7] Rush AJ , Aaronson ST , Demyttenaere K. Difficult-to-treat depression: a clinical and research roadmap for when remission is elusive. Aust N Z J Psychiatry. 2019;53:109–18. doi:10.1177/0004867418808585. 30378447

[8] Gaynes BN , Lux L , Gartlehner G , Asher G , Forman-Hoffman V , Green J , et al. Defining treatment-resistant depression. Depress Anxiety. 2020;37:134–45. doi:10.1002/DA.22968. 31638723

[9] Malhi GS , Irwin L , Hamilton A , Morris G , Boyce P , Mulder R, et al. Modeling mood disorders: an ACE solution? Bipolar Disord. 2018;20:4–16. doi:10.1111/BDI.12700. 30328224

[10] Fava GA , Cosci F , Guidi J , Rafanelli C. The deceptive manifestations of treatment resistance in depression: a new look at the problem. Psychother Psychosom. 2020;89:265–73. doi:10.1159/000507227. 32325457

[11] Malhi GS , Parker GB , Crawford J , Wilhelm K , Mitchell PB . Treatment-resistant depression: resistant to definition? Acta Psychiatr Scand. 2005;112:302–9. doi:10.1111/J.1600-0447.2005.00602.X. 16156838

